# Comparative Evaluation of Push-Out Bond Strength of Glass Fiber Posts and Carbon Fiber Posts in Root Canals Treated With Calcium Hydroxide–Based, Resin-Based, and Bioceramic-Based Root Canal Sealers: Protocol for an In Vitro Study

**DOI:** 10.2196/76621

**Published:** 2026-01-26

**Authors:** Vinus Shivlani, Manoj Chandak, Sanika Damle

**Affiliations:** 1 Department of Conservative Dentistry and Endodontics Faculty of Dentistry Datta Meghe Institute of Medical Sciences Wardha, Maharashtra India

**Keywords:** bioceramic sealer, endodontic sealer, fiber post, carbon post, resin-based sealer, calcium hydroxide–based sealer

## Abstract

**Background:**

For root canal procedures to be successful, adequate bond strength between endodontic sealer and post material is necessary. For postendodontic restorations, glass fiber posts and carbon fiber posts are frequently used. Depending on the type of root canal sealer used, such posts may work differently. The chemical composition and characteristics of calcium hydroxide–based, resin-based, and bioceramic-based sealers vary, which may have an impact on the posts’ binding strength. Therefore, optimizing rehabilitative results requires an understanding of these connections.

**Objective:**

This study aims to evaluate and compare the push-out bond strength of glass fiber posts and carbon fiber posts in root canals treated with calcium hydroxide–based, resin-based, and bioceramic-based endodontic sealers.

**Methods:**

A total of 60 extracted human premolars will be used in this in vitro investigation. After canal preparation, specimens will be separated into 3 groups according to the type of sealer used (bioceramic-based, resin-based, and calcium hydroxide–based). The type of post (carbon fiber or glass fiber) will be used to further split each group into 2 subgroups. A universal testing machine will be used to exert a compressive force on each post to test its push-out bond strength. Bond strength data will be recorded in megapascals and analyzed using ANOVA and post hoc tests.

**Results:**

The results are expected to demonstrate significant differences in push-out bond strength among different post and sealer combinations. Glass fiber posts are expected to have higher bond strength values than carbon fiber posts across all sealer groups, with the highest bond strength anticipated in the bioceramic-based sealer group. Resin-based sealers are expected to exhibit intermediate bond strength values, whereas calcium hydroxide–based sealers are expected to show the lowest bond strength values across both types of post.

**Conclusions:**

Glass fiber posts are expected to offer superior push-out bond strength in comparison to carbon fiber posts, especially when used with bioceramic-based sealers. The type of root canal sealer significantly affects bond strength, with bioceramic-based sealers providing the most reliable bond. Findings are expected that will suggest that careful selection of both post material and sealer type is necessary to enhance the long-term success of root canal restorations.

**International Registered Report Identifier (IRRID):**

DERR1-10.2196/76621

## Introduction

### Background

The resistance of a tooth to fracture should be enhanced by restoration. Posts are indicated to preserve the core that restores lost coronal structure in teeth with significant damage [1]. When the remaining cervical tooth tissue is unable to hold and retain the tooth, a post should be considered. In recent decades, the most popular use of metal cast posts has been for post and core restorations [2]. However, conventional cast posts have several drawbacks, including stiffness due to their high modulus of elasticity compared with that of dentin and the need for several fabrication sessions.

Concerns such as root fracture, risk of corrosion, and loss of retention of the post or crown associated with different metals prompted the search for posts with better load-bearing and retentive properties. According to clinical research [3], teeth rebuilt with fiber-reinforced posts have a success rate of 95% to 99%, with no root fractures occurring during the study period. The main benefits of fiber posts include high fatigue and tensile strength, a modulus of elasticity similar to that of dentin, and the ability to be cemented with an adhesive luting material, which reduces friction between the post and root canal walls and ensures uniform distribution of applied forces along the length of the post [3].

The milestone that altered some of the underlying concepts in the restoration of teeth that had undergone endodontic treatment was the introduction of the first fiber post in dentistry, known as carbon fiber–reinforced resin post (or carbon fiber post), by Duret et al [4]. These posts were the first widely accepted substitute for prefabricated metal and cast posts. Initially, unidirectional carbon or graphite fibers with a diameter of 8 μm were used to strengthen a matrix composed of 64% epoxy resin by weight in carbon fiber posts. Nevertheless, as carbon fiber posts are dark in color, they lack esthetic appeal. Their strong flexural resistance means that less dentin structure needs to be removed, which helps prevent fracture of both the post and the remaining tooth [4]. When subjected to intense forces, the post can follow tooth movement because its modulus of elasticity is equal to that of dentine, thereby providing weakened roots with greater resistance to breakage [4].

To improve the distribution of masticatory pressures and ensure more retention and support for restorative materials, glass fiber posts are recommended for the rehabilitation of severely damaged teeth. The benefits of glass fiber posts include high tensile strength, esthetic appeal, and a dentin-like modulus of elasticity, which distributes stress uniformly along the length of the post [5]. However, comparative research indicates that carbon fiber posts exhibit more flexural resistance than glass fiber posts [6]. Posts are frequently bonded to root canal walls using resin cements. Fiber posts adhere to dentin and resin cements with remarkable bond strength.

Although several types of prefabricated posts made of metal and resin are currently available in the market, carbon fiber posts and glass fiber posts are often advised because they strengthen the tooth structure and increase its overall integrity. Previous studies provide valuable insights into the performance of different post and sealer combinations under stress, mimicking the conditions encountered during functional loading [7]. Furthermore, the longevity and adherence of a post retainer to radicular dentin may be affected by the endodontic sealer, depending on its composition. Presence of eugenol in the cement’s composition significantly reduced adhesion, according to research showing the effect of zinc oxide–eugenol–based cement on intraradicular post retention [7]. The effects of zinc oxide–eugenol cement, epoxy resin, and calcium hydroxide on the retention of prefabricated titanium dowels luted with resin cement were investigated in a different investigation. According to the results [8], the groups that did not use cement and those that used a sealant containing eugenol differed significantly. In a related investigation, Ngoh et al [9] found that eugenol decreased the resin cement’s ability to adhere to dentin in the coronal third of the root.

In clinical practice, calcium hydroxide–based, resin-based, and bioceramic-based root canal sealers are commonly used, although a variety of sealers have been developed and are commercially available. Although resin-based sealers are well known for their superior bonding powers to dentin and root posts, calcium hydroxide is recognized for its antibacterial properties and its capacity to aid in the healing of periapical tissues. Bioactivity, biocompatibility, and sealing properties of bioceramic sealers, a more recent class of materials, are valued [10]. This emphasizes the necessity of assessing the effects of various fiber posts and popular sealers combinations on bond strength in endodontically treated teeth.

Due to varying properties such as surface chemistry and elasticity of carbon fiber posts and glass fiber posts, along with resin-based, bioceramic-based, and calcium hydroxide–based sealers used in endodontics, these materials leave behind a distinct residual impact on dentin, which ultimately affects the adhesion of resin cements. Hence, studying all these combinations would suggest a more reliable post and sealer pairing, maximizing push-out bond strength and guiding toward better clinical restorability. For restorations retaining the dental post to be successful over the long term, a good relationship between post materials and root canal sealers is essential. Few studies have examined the effects of different endodontic sealers on the bonding interface between the post, resin cement, and radicular dentin, although the mechanical compatibility of fiber posts with dentin has been extensively researched. It is well known that some sealers, especially those based on eugenol, inhibit the polymerization of resin cements, thereby compromising post retention [7-9].

Although bioceramic sealers are valued for their bioactivity and sealing ability and differ chemically from other sealers, resin-based sealers provide better adhesion to dentin and greater compatibility with resin cements [10]. The surface effects and residual chemical composition of these sealers on canal walls can differ greatly, which affects how well they adhere to glass fiber posts and carbon fiber posts. Nevertheless, there are currently no thorough analyses in the literature contrasting the push-out bond strength of these fiber post types with various widely used sealers.

There is a dearth of literature comparing the bond strength of carbon fiber posts and glass fiber posts in root canals treated with calcium hydroxide–based, resin-based, or bioceramic-based endodontic sealers [10].

### Objectives

The primary objective of this study is to evaluate the push-out bond strength of glass fiber posts and carbon fiber posts in root canals treated with calcium hydroxide–based, resin-based, and bioceramic-based sealers.

Moreover, this study also compares the push-out bond strength of glass fiber posts and carbon fiber posts when used with the same root canal sealers.

## Methods

### Materials Required

A total of 60 extracted human premolars will be chosen for this investigation. Teeth that are removed for orthodontic reasons and are devoid of cavities, cracks, or previous endodontic procedures will be used. The teeth will be preserved in distilled water after being cleaned with ultrasonic scalers to remove any calculus and soft tissue remnants before the experiment. The teeth will be decoronated at 15 (SD 1) mm using water coolant and slow-speed disks.

A high-speed handpiece (KaVo) equipped with a round carbide bur (size #2) will be used to create a straight-line access to the canals. After that, a crown-down procedure will be used to instrument the root canals. To clean the canal and remove the debris, 3% sodium hypochlorite will be used.

The first cleaning will be done with a size 15 K-file (Dentsply Sirona). Using step-back instrumentation, the canal will be progressively enlarged up to a size 40 file. There will be extensive irrigation between each file to get rid of germs and debris. A size 40/04 rotary file (ProTaper Next, Dentsply Sirona) will be used for the final preparation once the canals reach the necessary size. This will guarantee that the canals are correctly tapered and well shaped. After removal of the smear layer with final irrigation using a solution of 3% sodium hypochlorite and 17% ethylenediaminetetraacetic acid, the canals will be rinsed with distilled water.

After preparation, the following 3 distinct types of root canal sealers will be used to obturate the canals:

1. Calcium hydroxide–based sealer (Sealapex, Kerr)
2. Resin-based sealer (Meta Adseal, Meta Biomed)
3. Bioceramic-based sealer (Bio C sealer, Angelus) The root canals will be ready for post insertion once the sealer has solidified, which should happen in 6 to 7 days. To make room for the placement of posts, a little quantity of the obturation material will be removed from the canals using a Gates-Glidden drill (Dentsply Sirona). Using a bespoke drill, a uniform post space of approximately 10 to 12 mm will be created.

### Luting and Post Selection

This study will use carbon fiber posts (Reforpost, Angelus) and glass fiber posts (Reforpost, Angelus). To match the designated post space, the posts will be chosen according to their individual diameters. To improve bonding to the sealer, the posts will be coated with a silane coupling agent (Silane, 3M ESPE) before being placed.

### Cementing

In accordance with the manufacturer’s instructions, a self-etch resin cement (Dentsply, Calibra, Universal Self-Adhesive resin system) will be used to cement the posts within the post space. To ensure that the sealer is evenly distributed around the post, the posts will be carefully placed into the prepared canals. After removing any excess cement, the posts will be light-cured from various angles for 20 seconds each to ensure full polymerization. It is recommended to apply the standardized force of 20 to 30 N for cementation of the post to the root dentin. While light curing, the tip of the curing unit must be placed at 90° to the canal, ensuring maximum contact and light exposure.

### Push-Out Bond Strength Test

To allow the cement to adequately set, these specimens will be kept in distilled water at 37 °C for 1 day following the post luting procedure. A diamond blade in a water-cooled cutting machine will next be used to segment the teeth transversely into slices that are 1 mm thick. Once the slices are placed in acrylic blocks, a universal testing machine will be used to measure the push-out bond strength at a crosshead speed of 1 mm per minute. The post will be compressed in an apical-coronal orientation using a cylindrical rod until dislodgement occurs.

The bond strength will be estimated by dividing the maximum force required for post dislodgement by the surface area of the post.

### Inclusion Criteria

Only healthy human extracted teeth, free of caries, fractures, and any prior dental procedures, will be included in the study. Teeth must have enough coronal structure for post placement, a single straight root canal system, and a root length of approximately 14 mm. Teeth without previous endodontic treatment and extracted for nonpathological reasons, such as orthodontics, will be used.

### Exclusion Criteria

Caries-laden, fractured, cracked, or structurally flawed teeth will not be included. Additionally, teeth with numerous roots or intricate canal systems, previous endodontic procedures, severe root resorption, or inadequate root length (<12 mm) will be excluded. Teeth with inadequate coronal structure that cannot sustain appropriate post implantation will be excluded.

### Sample Size

The sample size formula is as follows:

n_1_=Δ2(σ_1_^2^+κσ_2_^2^) (Z_1−α/2_+Z_1−β_)2

Where n_1_=sample size of group 1; n_2_=sample size of group 2; σ_1_=SD of group 1; σ_2_=SD of group 2; Δ=difference in group means; κ=ratio of n_2_ to n_1_ (n_2_/n_1_); Z_1−α/2_=2-sided Z value (eg, Z=1.96 for 95% CI); and Z_1−β_=power.

Specific values are as folows: mean bond strength in Endofill (Dentsply Sirona)+Variolink II=10.38; mean bond strength in Endofill+RetyXU200=11.75; σ_1_=SD of bond strength in Endofill+Variolink II=1.8; and σ_2_=SD of bond strength in Endofill+RetyXU200=2.0.

Thus, for detecting a mean difference of 1.37, that is, Δ=11.75−10.38=1.37, we need K=1; N=(1.8 × 1.8 + 2.0 × 2.0) (1.9 + 0.7)^2^ 2.0 × 2.0 = 9.87 = 10 specimens in each group (6 × 10 = 60). The power of the test will be 80% and the level of significance will be 5% (95% CI).

### Sample Allocation

A total of 60 samples will be divided into 2 main groups based on the type of post: 30 samples each for carbon fiber posts (group A) and glass fiber posts (group B). Each group will then be divided into 3 subgroups according to the type of root canal sealer used: calcium hydroxide–based, resin-based, or bioceramic-based.

All 60 samples will first be allocated to a specific post type (n=30 each). The segregation of samples according to different sealer types (n=10 each) will be done using a random sequence. This allocation will be sealed and sequentially numbered by an independent researcher. Outcome assessment of push-out bond strength will be performed by a blinded evaluator using neural-coded samples, thereby minimizing detection bias and ensuring unbiased allocation. This study will use a comparative design: an in vitro experimental study comparing multiple intervention groups under standardized conditions.

### Ethical Considerations

Ethics approval for this study has been obtained from the institutional ethics committee of Datta Meghe Institute of Higher Education & Research (IEC/2025/544).

All extracted teeth will be collected from the department of oral surgery, ensuring that they are not at risk of harm due to the use of the teeth in research. This study will follow strict guidelines for ethical handling of human tissues. All study data will be handled in accordance with applicable data protection and privacy regulations. Participant confidentiality will be strictly maintained throughout the study. Personal identifiers will be removed and replaced with unique study codes prior to data analysis. The key linking participant identities to study codes will be stored separately and securely, accessible only to the principal investigator.

Electronic data will be stored on password-protected computers and/or encrypted institutional servers, while any physical records will be kept in locked cabinets within secure research facilities. Only authorized members of the research team will have access to the data. No individually identifiable information will be disclosed in any publications, presentations, or reports arising from this study.

Written informed consent will be obtained from all participants prior to enrollment in the study. Participants will be provided with detailed information regarding the study objectives, procedures, potential risks and benefits, data handling practices, and their right to withdraw from the study at any time without any impact on their care. For participants who are unable to provide written consent, consent will be obtained from a legally authorized representative in accordance with institutional and ethical guidelines

### Primary Outcome

The primary finding of this study is expected to be that glass fiber posts will exhibit the strongest push-out bond, particularly when combined with resin-based and bioceramic-based sealers. Conversely, it is expected that carbon fiber posts will exhibit a lower push-out bond strength, especially when combined with calcium hydroxide–based sealers.

### Statistical Analysis Plan

All push‑out bond strength values will first be summarized using descriptive statistics (mean [SD] for each post and sealer combination). Data distribution normality and variance homogeneity will be assessed using the Shapiro-Wilk and Levene tests, respectively. If both assumptions are satisfied, group comparisons will be performed using one‑way ANOVA, followed by Tukey post hoc tests for multiple comparisons and independent Student *t* tests (2-tailed) for paired contrasts. If variance homogeneity is violated, Welch ANOVA (or Brown-Forsythe test) will be used, whereas if normality assumptions are not met—especially in small or skewed samples—data transformation (eg, logarithmic or square root) or nonparametric alternatives will be used, including the Mann-Whitney U test for 2-group comparisons and the Kruskal-Wallis test for comparisons involving ≥3 groups, with appropriate pairwise follow-up testing (eg, Dunn test). All analyses will be conducted using SPSS (version 27.0; IBM Corp) and GraphPad Prism (version 7.0; GraphPad Software Inc) to ensure robustness in handling assumption violations while preserving statistical power.

## Results

### Luting and Post Selection

Nearly all posts (95%-98%) are expected to fit within +0.1 or −0.1 mm of the designated post spaces, ensuring appropriate seating. Under microscopy, mixed failures are anticipated in approximately 60% of specimens, with adhesive failures at the cement-dentin interface comprising approximately 30% and adhesive failures at the cement-post interface comprising approximately 10%.

### Cementing

Posts are expected to seat fully under a standardized force of 20 to 30 N without delay. Light curing at a 90° angle is expected to yield appropriate polymerization beyond International Organization for Standardization (ISO) standards. Cement extrusion beyond the apex is anticipated in approximately 5% of specimens, occurring most frequently in the calcium hydroxide–based sealer (Sealapex) group.

### Push-Out Bond Strength

Results are expected to demonstrate substantial differences in push-out bond strength among the different post and sealer combinations. Glass fiber posts are expected to have higher bond strength values than carbon fiber posts across all sealer groups, with the highest bond strength observed in the bioceramic-based sealer group. Resin-based sealers are expected to show intermediate bond strengths, whereas calcium hydroxide–based sealers are expected to show the lowest bond strength across both types of posts.

### Timeline

The expected results of this study will be published by January 2026. This research project is currently not funded. Data collection is underway, and analysis of the results began in August 2025.

## Discussion

The restoration of endodontically treated teeth using posts has gained immense popularity and is proven to provide better clinical results in badly damaged teeth. Restoration of a tooth with severely damaged coronal structure has been a challenge for endodontists. Effective postendodontic restoration is frequently necessary for long-term success of endodontically treated teeth, and fiber-reinforced posts have become attractive options because of their biomechanical compatibility with dentin. The 2 most commonly used types, glass fiber posts and carbon fiber posts, have unique physical and chemical properties that affect their adhesion to the root dentin.

In root canal obturation, a variety of sealers with differing properties have been used. Biocompatibility and potent antimicrobial effects are the main criteria in the development of root canal sealers [10,11]. Additionally, the bond strength of posts to root dentin is considered to be better in nonfilled canals than that in filled canals [10]. Hence, the most appropriate sealer must be used in endodontic treatment because the type of sealer used can severely affect the bond strength of posts to root dentin. With regard to all these concepts, the effect of different sealer types affecting the push-out bond strength of glass fiber posts and carbon fiber posts to root dentin will be studied.

Teixeira et al [12] analyzed the effect of various endodontic sealers—EndoFill, Sealapex, and EndoREZ (Ultradent Products Inc)—on the bond strength of carbon fiber posts cemented with resin cement. A total of 30 extracted human premolars were segregated based on sealer type and post cementation timing (48 hours vs 7 days) [13]. The results showed that EndoREZ provided the highest bond strength, particularly in the coronal and middle thirds, whereas EndoFill showed the lowest. Mixed failure modes were expected to be the most common. The study emphasized that sealer type, especially the use of EndoREZ, significantly influences bond strength in post retained restorations [12].

A study by Forough Reyhani et al [14] assessed the effect of various endodontic sealers, including MTA Fillapex (Angelus), Dorifill, and AH Plus (Dentsply DeTrey GmbH), on the bond strength of fiber posts cemented with a self-etch adhesive. A total of 72 upper incisors were split up into 4 groups, each of which will be filled with gutta-percha and allocated to either AH Plus, Dorifill, MTA Fillapex, or a control group (no sealer). According to the results, the control group had the highest bond strength (mean 4.45, SD 0.09 MPa), whereas Dorifill had the lowest (mean 1.02, SD 0.03 MPa). Although MTA Fillapex and AH Plus demonstrated stronger bonds than Dorifill, their values remained lower than those of the control group. These findings suggest that the type of sealer significantly influences the bond strength of fiber posts, with MTA Fillapex exhibiting lower resistance to post dislodgment than other sealers [14].

Ruiz et al [13] investigated the influence of root canal sealers, storage duration, and cementation systems on bond strength by dividing 56 extracted human canines into 8 groups. Specimens were sectioned and subjected to bond strength testing after designated storage periods [13]. Their findings revealed that only the storage duration following obturation had a significant impact on bond strength, whereas the type of sealer and cementation system did not show a statistically significant effect (*P*>.05). Notably, glass fiber posts cemented 6 months after obturation demonstrated higher bond strength than those cemented after 1 week, suggesting that the timing of cementation plays a more critical role than the type of sealer used [13].

This study has certain limitations, including in vitro conditions that do not fully replicate the oral environment, the use of premolar teeth that may not represent all tooth types, and a short-term evaluation that does not account for long-term factors such as wear or cyclic loading. Material variability and the lack of clinical follow-up further limit the generalizability of the results. Additionally, the sample size may be insufficient to detect small differences between groups, especially when effect sizes are minimal.

The objective of this study is to assess the effects of various post types and root canal sealers on the push-out bond strength of endodontically treated teeth. The research will yield important information for enhancing restorative techniques and extending the durability of endodontic procedures.

## Abbreviations

ISO: International Organization for Standardization

## Data Availability

The datasets generated or analyzed during this study are available from the corresponding author on reasonable request, subject to ethical and data protection restrictions.
